# Supplementary material for: Individual differences in avoiding feelings of disgust: Development and construct validity of the disgust avoidance questionnaire
Source: PLoS One. 2021 Mar 10;16(3):e0248219. doi: 10.1371/journal.pone.0248219 (PMC7946286; doi:10.1371/journal.pone.0248219)
Supplement: S1 Table — (DOCX) [file pone.0248219.s001.docx]

| **Table S1. Items of the MEAQ, EAQ, and CAQ Used as Source Items for the DAQ** | | |
| --- | --- | --- |
|  | **Source Item (ordered by Scale)** | **DAQ Item** |
| ***MEAQ*** | |  |
|  | 1. I won’t do something if I think it will make me uncomfortable | 3. I won’t do something if I know it will be revolting. |
|  | 3. When something upsetting comes up, I try very hard to stop thinking about it | 14. When thoughts about repulsive things come up, I try very hard to stop thinking about them. |
|  | 9. When negative thoughts come up, I try to fill my head with something else | 24. When thoughts about revolting things come up, I try to fill my head with something else. |
|  | 14. I rarely do something if there is a chance that it will upset me | 1. I rarely do something if there is a chance that it will disgust me. |
|  | 15. I usually try to distract myself when I feel something painful | 20. I usually try to distract myself when I feel disgusted. |
|  | 20. I work hard to avoid situations that might bring up unpleasant thoughts and feelings in me | 9. I try hard to avoid situations that might bring up feelings of repulsion in me. |
|  | 22. When upsetting memories come up, I try to focus on other things | 22. When memories of disgusting experiences come up, I try to focus on other things. |
|  | 39. If I am starting to feel trapped, I leave the situation immediately | 8. If I am in a situation in which I feel revolted, I leave the situation immediately. |
|  | 55. I avoid situations if there is a chance that I’ll feel nervous | 5. I try to avoid activities that could make me feel disgusted.  13. I avoid situations if there is a chance that I will feel revolted.  15. I avoid objects that can trigger feelings of disgust. |
|  | 59. I’m quick to leave any situation that makes me feel uneasy | 2. I am quick to stop any activity that makes me feel disgusted.  10. I am quick to leave any situation that makes me feel disgusted. |
| ***EAQ*** | |  |
|  | 1. If I start feeling strong positive emotions, I prefer to leave the situation. | 4. If I am doing something that makes me feel repulsion, I prefer to stop that activity.  6. If I start feeling strong disgust, I prefer to leave the situation. |
|  | 3. When happy thoughts occur to me, I push them out of my mind. | 12. When I think about something gross, I push those thoughts out of my mind. |
|  | 16. If sad thoughts cross my mind, I try to push them away as much as possible. | 16. If thoughts about disgusting things cross my mind, I try to push them away as much as possible. |
|  | 20. If I feel or think something unpleasant, I try to wipe it out or distract myself. | 18. If I feel disgusted or think about something repulsive, I try to distract myself. |
| ***CAQ*** | |  |
|  | 2. I avoid certain situations that lead me to pay attention to things I do not want to think about. | 11. I avoid certain situations that make me pay attention to disgusting things. |
|  | 6. I try not to think about the most upsetting aspects of some situations so as not to be too afraid. | 19. I try not to think about gross situations.  21. I try hard to avoid thinking about a repulsive past situation. |
|  | 8. I distract myself to avoid thinking about certain disturbing subjects. | 23. I distract myself to avoid thinking about things that disgust me. |
|  | 13. To avoid thinking about subjects that upset me, I force myself to think about something else. | 25. To avoid thinking about things that revolt me, I force myself to think about something else. |
|  | 18. I avoid actions that remind me of things I do not want to think about. | 7. I avoid actions that remind me of repulsive things. |
|  | 22. I avoid situations that involve people who make me think about unpleasant things. | 17. I avoid places that make me think of things that disgust me. |
| Note. *MEAQ = Multidimensional Experiential Avoidance Questionnaire; EAQ = Experiential Avoidance Questionnaire; CAQ = Cognitive Avoidance Questionnaire* | | |
