# Supplementary material for: Individual differences in avoiding feelings of disgust: Development and construct validity of the disgust avoidance questionnaire
Source: PLoS One. 2021 Mar 10;16(3):e0248219. doi: 10.1371/journal.pone.0248219 (PMC7946286; doi:10.1371/journal.pone.0248219)
Supplement: S3 Table — (DOCX) [file pone.0248219.s003.docx]

| **Table S5. Two-Factor EFA on the Escape-Focused Items (Initial Item Set).** | | |
| --- | --- | --- |
| **Items** | **Two-factor EFA**  (CFI = .955; RMSEA = .182) | |
|  | **1** | **2** |
| **Disgust Escape** |  |  |
| 1. I am quick to stop any activity that makes me feel disgusted. | **.78** | .03 |
| 2. If I am doing something that makes me feel repulsion, I prefer to stop that activity. | **.79** | -.05 |
| 3. If I start feeling strong disgust, I prefer to leave the situation. | **.77** | .03 |
| 4. If I am in a situation in which I feel revolted, I leave the situation immediately. | **.79** | .03 |
| 5. I am quick to leave any situation that makes me feel disgusted. | **.91** | -.01 |
| 6. When I think about something gross, I push those thoughts out of my mind. | .07 | **.77** |
| 7. When thoughts about repulsive things come up, I try very hard to stop thinking about them. | .02 | **.85** |
| 8. If thoughts about disgusting things cross my mind, I try to push them away as much as possible. | .07 | **.86** |
| 9. If I feel disgusted or think about something repulsive, I try to distract myself. | -.00 | **.89** |
| 10. I usually try to distract myself when I feel disgusted. | -.06 | **.90** |
| 11. When memories of disgusting experiences come up, I try to focus on other things. | -.00 | **.80** |
| 12. When thoughts about revolting things come up, I try to fill my head with something else. | -.05 | **.91** |

*Note*. Factor loadings of ≥ 0.3 are marked bold.
