# Supplementary material for: Individual differences in avoiding feelings of disgust: Development and construct validity of the disgust avoidance questionnaire
Source: PLoS One. 2021 Mar 10;16(3):e0248219. doi: 10.1371/journal.pone.0248219 (PMC7946286; doi:10.1371/journal.pone.0248219)
Supplement: S1 Appendix — (DOCX) [file pone.0248219.s004.docx]

**S2 Appendix**

**The Initial Item Set (25) of the Disgust Avoidance Questionnaire (DAQ)**

| **[Instruction**:] This questionnaire will assess how people cope with situations or activities that can elicit disgust, for example: coming into contact with bodily fluids of another person, accidentally eating rotting food, seeing mutilated bodies on the TV, having sexual contact with someone you are not attracted to, hearing about incest, witnessing dehumanization or harm done to others.  For each of the statements presented below, please indicate the extent to which you agree or disagree with the statements. | | | | | | | |
| --- | --- | --- | --- | --- | --- | --- | --- |
|  | (1)  Strongly disagree | (2)  Disagree | (3)  Somewhat disagree | (4)  Neither agree nor disagree | (5)  Somewhat agree | (6)  Agree | (7)  Strongly agree |
| 1. I rarely do something if there is a chance that it will disgust me.* |  |  |  |  |  |  |  |
| 2. I am quick to stop any activity that makes me feel disgusted. |  |  |  |  |  |  |  |
| 3. I won’t do something if I know it will be revolting.* |  |  |  |  |  |  |  |
| 4. If I am doing something that makes me feel repulsion, I prefer to stop that activity.* |  |  |  |  |  |  |  |
| 5. I try to avoid activities that could make me feel disgusted. |  |  |  |  |  |  |  |
| 6. If I start feeling strong disgust, I prefer to leave the situation. |  |  |  |  |  |  |  |
| 7. I avoid actions that remind me of repulsive things. |  |  |  |  |  |  |  |
| 8. If I am in a situation in which I feel revolted, I leave the situation immediately. |  |  |  |  |  |  |  |
| 9. I try hard to avoid situations that might bring up feelings of repulsion in me. |  |  |  |  |  |  |  |
| 10. I am quick to leave any situation that makes me feel disgusted. |  |  |  |  |  |  |  |
| 11. I avoid certain situations that make me pay attention to disgusting things. |  |  |  |  |  |  |  |
| 12. When I think about something gross, I push those thoughts out of my mind. |  |  |  |  |  |  |  |
| 13. I avoid situations if there is a chance that I will feel revolted.* |  |  |  |  |  |  |  |
| 14. When thoughts about repulsive things come up, I try very hard to stop thinking about them. |  |  |  |  |  |  |  |
| 15. I avoid objects that can trigger feelings of disgust.* |  |  |  |  |  |  |  |
| 16. If thoughts about disgusting things cross my mind, I try to push them away as much as possible. |  |  |  |  |  |  |  |
| 17. I avoid places that make me think of things that disgust me. |  |  |  |  |  |  |  |
| 18. If I feel disgusted or think about something repulsive, I try to distract myself.* |  |  |  |  |  |  |  |
| 19. I try not to think about gross situations. |  |  |  |  |  |  |  |
| 20. I usually try to distract myself when I feel disgusted.* |  |  |  |  |  |  |  |
| 21. I try hard to avoid thinking about a repulsive past situation. |  |  |  |  |  |  |  |
| 22. When memories of disgusting experiences come up, I try to focus on other things.* |  |  |  |  |  |  |  |
| [23.] This is a control question. Please tick the left-most answer option. |  |  |  |  |  |  |  |
| 23. I distract myself to avoid thinking about things that disgust me. |  |  |  |  |  |  |  |
| 24. When thoughts about revolting things come up, I try to fill my head with something else. |  |  |  |  |  |  |  |
| 25. To avoid thinking about things that revolt me, I force myself to think about something else. |  |  |  |  |  |  |  |
| *Note*. This is the item order that was presented to the participants, item numbers in this Table thus deviate from the item numbers specified above.  *The asterisk denotes items that were excluded in the item-reduction (step 1) | | | | | | | |
