# Supplementary material for: Individual differences in avoiding feelings of disgust: Development and construct validity of the disgust avoidance questionnaire
Source: PLoS One. 2021 Mar 10;16(3):e0248219. doi: 10.1371/journal.pone.0248219 (PMC7946286; doi:10.1371/journal.pone.0248219)
Supplement: S2 Appendix — (DOCX) [file pone.0248219.s005.docx]

**S3 Appendix**

**The Reduced Item Set (17) and Subscale Calculation of the Disgust Avoidance Questionnaire (DAQ)**

| **[Instruction**:] This questionnaire will assess how people cope with situations or activities that can elicit disgust, for example: coming into contact with bodily fluids of another person, accidentally eating rotting food, seeing mutilated bodies on the TV, having sexual contact with someone you are not attracted to, witnessing dehumanization or harm done to others^a^.  For each of the statements presented below, please indicate the extent to which you agree or disagree with the statements. | | | | | | | |
| --- | --- | --- | --- | --- | --- | --- | --- |
|  | (1)  Strongly disagree | (2)  Disagree | (3)  Somewhat disagree | (4)  Neither agree nor disagree | (5)  Somewhat agree | (6)  Agree | (7)  Strongly agree |
| 1. I try to avoid activities that could make me feel disgusted. |  |  |  |  |  |  |  |
| 2. When I think about something gross, I push those thoughts out of my mind. |  |  |  |  |  |  |  |
| 3. I am quick to stop any activity that makes me feel disgusted. |  |  |  |  |  |  |  |
| 4. I try not to think about gross situations. |  |  |  |  |  |  |  |
| 5. When thoughts about repulsive things come up, I try very hard to stop thinking about them. |  |  |  |  |  |  |  |
| 6. I avoid actions that remind me of repulsive things. |  |  |  |  |  |  |  |
| 7. If I start feeling strong disgust, I prefer to leave the situation. |  |  |  |  |  |  |  |
| 8. If thoughts about disgusting things cross my mind, I try to push them away as much as possible. |  |  |  |  |  |  |  |
| 9. I try hard to avoid thinking about a repulsive past situation. |  |  |  |  |  |  |  |
| 10. I try hard to avoid situations that might bring up feelings of repulsion in me. |  |  |  |  |  |  |  |
| 11. If I am in a situation in which I feel revolted, I leave the situation immediately. |  |  |  |  |  |  |  |
| 12. When thoughts about revolting things come up, I try to fill my head with something else. |  |  |  |  |  |  |  |
| 13. I avoid certain situations that make me pay attention to disgusting things. |  |  |  |  |  |  |  |
| 14. I distract myself to avoid thinking about things that disgust me. |  |  |  |  |  |  |  |
| 15. I am quick to leave any situation that makes me feel disgusted. |  |  |  |  |  |  |  |
| 16. I avoid objects that can trigger feelings of disgust. |  |  |  |  |  |  |  |
| 17. To avoid thinking about things that revolt me, I force myself to think about something else. |  |  |  |  |  |  |  |
| *Note*. This is the item order that was presented to the participants, item numbers in this Table thus deviate from the item numbers specified above.  ^a^ In the current project, the examples of disgust-elicitors included “hearing about incest”. We decided to omit this example from the final questionnaire because it may be considered as offensive. | | | | | | | |

**Subscale Calculation (unweighted sum scores):**

PREV: 1, 4, 6, 9, 10, 13, 14, 16, 17

BEH: 1, 3, 6, 7, 10, 11, 13, 15, 16

COG: 2, 4, 5, 8, 9, 12, 14, 17
