# Supplementary material for: Individual differences in avoiding feelings of disgust: Development and construct validity of the disgust avoidance questionnaire
Source: PLoS One. 2021 Mar 10;16(3):e0248219. doi: 10.1371/journal.pone.0248219 (PMC7946286; doi:10.1371/journal.pone.0248219)
Supplement: Attachment — Submitted filename: Response to Reviewers.docx [file pone.0248219.s007.docx]

Dear Dr. Federici and Reviewer 2,

On behalf of all authors, I hereby submit our further revised manuscript PONE- D-20-16240 "Individual Differences in Avoiding Feelings of Disgust: Development and Construct Validity of the Disgust Avoidance Questionnaire". We would like to thank you for evaluating our manuscript.

Below we describe in detail how we handled each of the remaining comments. Each comment is literally quoted, followed by our reply. Line references to the changes made to the manuscript refer to the *clean manuscript* (in which all track changes were accepted).

We hope that you will consider the comments to be adequately addressed and find the revised manuscript suitable for publication in PLOS ONE.

We look forward to hearing from you.

Sincerely, also on behalf of the other authors,

Paula von Spreckelsen

**Comment from the Editor:**

Although both Reviewers have appreciated the Authors’ responsiveness to their review and their efforts to revise the manuscript, one of them still found that the statistical analysis has not been performed appropriately. In particular, references and explanations for the measurement development approach should be provided.

We are happy to see that both reviewers valued our revisions to the manuscript. With regard to the statistical analyses, we provided additional explanations and references to the method in our response to the reviewer below and in the manuscript.

Before we turn to these changes, we would like to note that we adapted a small detail in the instruction of the DAQ (see S3 Appendix). In the instructions, we state:

“*This questionnaire will assess how people cope with situations or activities that can elicit disgust, for example: coming into contact with bodily fluids of another person, accidentally eating rotting food, seeing mutilated bodies on the TV, having sexual contact with someone you are not attracted to, hearing about incest, witnessing dehumanization or harm done to others.*

*For each of the statements presented below, please indicate the extent to which you agree or disagree with the statements.*”

We did not realize it at the time, but our example of a disgust elicitor “*hearing about incest*” may be considered offensive in some cultures. Because we do not wish to offend any culture, we thought it would be best to delete that example from the final questionnaire. We do not think that it would have an impact on our results, as we do not change any of the DAQ items, but merely omit one of the disgust-elicitors from the list of examples in the instructions. We adapted Supplementary material S3 by deleting the example and adding an explanation into the note of the Table.

**Comments by Reviewer 2:**

Although I appreciate the authors’ responsiveness to my initial review and their efforts to revise the manuscript, I am still not convinced that this disgust avoidance measure is needed. As the authors have argued that this measure may be especially important for understanding psychopathology, evidence of this is necessary. In particular, it seems crucial to demonstrate that the disgust avoidance measure has predictive validity unique to or above and beyond experiential avoidance measures.

We are happy that the reviewer appreciates our revisions. We firstly would like to say that we agree with the reviewer on the importance of examining the DAQ’s predictive validity. In our manuscript, we provide theoretical arguments about the DAQ’s potential relevance to the field of clinical psychology and voice that this potential relevance remains to be examined in future research (e.g., lines 37 – 39; lines 698 - 699; lines 729 – 731; lines 748 - 750; lines 779 – 787). Because we do not claim to have established the clinical relevance of the DAQ in the current project, we do not agree that it is crucial for the current paper to demonstrate said clinical relevance/predictive validity. The goal of the current manuscript is to introduce and to share our measure of disgust avoidance with the research community. We regard the project as a starting point for research into this subject with future research continuing to examine and evaluate its merit in psychopathology and possibly other fields of psychology (e.g., social psychology). It is common for questionnaires to be re-examined, optimized, and revised, with the goal to refine their validities and test their application and relevance to different domains. We therefore advocate that the judgement of the potential relevance or necessity of the DAQ is to be left to the research community.

The stepwise approach to item reduction is unusual. Standard practice would be to conduct a series of EFAs with all of the items to identify factor structure and reduce items. I can’t say that I’ve seen a measurement development paper in which separate EFAs were conducted with each set of factor items. References for this approach should be provided and an explanation for why this approach, rather than standard approach, was taken. I still question the four-factor solution with items that cross-load. I would be curious to know if the factor structure and final set of items differ if a standard approach was utilized.

We understand the reviewer’s concerns and would like to offer a more detailed explanation for the method which we chose to select the item pool of the DAQ in Study 1. It is indeed common to run an EFA on all items of a questionnaire when the goal is factor extraction. Our goals for the analyses in Study 1 were two-fold: Step 1) Item selection (EFAs on item sets; main goal); Step 2) ‘Factor extraction’ (EFA on all items; secondary goal). We decided on that order because our goal was to first create a proper item set before evaluating examining the factor structure (e.g., suboptimal/problematic items could have influenced the results of a factor extraction). The EFAs on the item-sets were therefore part of the item selection, which can be based both on statistical and/or ‘judgmental’ criteria (e.g., [1]), but were not part of a ‘factor extraction’. Item selection is part of any questionnaire development. We simply included it as a statistical step in our manuscript, which might have led to some confusion.

As is done in item reduction (see [2] on their item reduction of facets of the JDI [Job Descriptive Index] using principal component analysis; see [3] describing the method of selecting items with the highest loading on a common factor underlying the items to guide item selection), we examined whether the items of one subscale loaded on one common factor. This step could have also been done in a CFA framework by using 1-factor CFA models. We decided to use EFA however because it allowed us to examine multi-factor models (next to 1-factor models) for a more exhaustive picture of item loadings. In other words, because our use of EFAs in this step was theory-driven, the distinction between EFA and CFA more or less vanished. After the item reduction, we then ran a 4-factor EFA to examine whether item loadings were in line with our hypothesized subscales.

We added a short introduction to the statistical approach we used in Study 1 (lines 226 - 235):

“*The main goal of Study 1 was to select items for the DAQ using both ‘judgmental’/evaluative (e.g., item content, wording, etc.) and statistical criteria (e.g., item loadings, reliability estimates; cf. [43]). We first compiled a list of potential items for the DAQ (based on judgmental criteria) and subsequently condensed it through a stepwise item reduction. The goal of the step-wise item reduction was to find a coherent item set per hypothesized subscale of the DAQ and it was based mainly on statistical criteria. More specifically, we used single- and multi-factor EFA (exploratory factor analysis; [44]) models and fitted them on the items of each hypothesized subscale to exclude ‘suboptimal’ items with the goal to create unidimensional factor models per subscale. As a last step, we fitted an EFA on all items to examine whether the item loadings were in line with our hypothesized subscales.”*

As we believe that the method we chose was suitable for item reduction, we have confidence in the final item sets of the DAQ. With regard to the final factor structure, we don’t see reasons for why it would have been different using a different methodology, because we see strong theoretical arguments for the overlapping factor structure. That being said, every methodology comes with limitations. In our case, we strongly based item reduction on statistical criteria (i.e., examining whether items loaded well on one common factor), which may have made our results more sample-dependent [3]. In addition, our method may also have resulted in a narrow item content [3]. However, our overall item selection was based on both statistical and judgmental criteria (the latter was used to compile the initial list of items & helped in item reduction for difficult cases), which may have somewhat protected us from these limitations. Nonetheless, the extent to which the findings generalize to other samples remains to be examined (which we added to our discussion).

We reflected on the potential limitations of our item selection methodology in the discussion section (lines 790 – 793):

“*Although we combined both judgmental and statistical criteria (e.g., in our item selection in Study 1) in order to decrease the sample-dependency of our results, it remains to be examined whether our results can be replicated in different samples.*”

Given the homogeneity of the two samples, I question the generalizability of these findings. Again, I doubt the utility of this measure. Additional data with more diverse samples is required.

We agree that more diverse samples are required to establish the general applicability of the DAQ. In our discussion section, we make explicit that future research is needed to establish the DAQ’s general applicability due to our restricted/homogeneous samples (lines 788 – 790). As we do not claim to have established a general applicability of the DAQ, we do not agree that it is crucial for the current paper to include additional data. As we already stated above, we regard the current paper as a starting point for future research refining its validity and generalizability.

However, in order to make it more explicit that future research into the generalizability of the DAQ is needed, we added the following to the abstract (lines 36 – 37):

“*In light of the exploratory nature of the project, future examinations of the DAQ’s validity and applicability to more diverse samples are essential*.”

**References**

1. Wieland A, Durach CF, Kembro J, Treiblmaier H. Statistical and judgmental criteria for scale purification. Supply Chain Management. 2017;22(4):321-328. doi: 10.1108/SCM-07-2016-0230
2. Stanton JM, Sinar EF, Balzer WK, Smith PC. Issues and strategies for reducing the length of self report scales. Pers. Psychol. 2002;55(1):167-194. doi: 10.1111/j.1744-6570.2002.tb00108.x
3. Widaman KF, Little T, Preacher KJ, Sawalani GM. On creating and using short forms of scales in secondary research. In: Trzesniewski KH, Donnellan MB, & Lucas RE, editors. Secondary data analysis: An introduction for psychologists. American Psychological Association. 2011. p. 39-61 doi: 10.1037/12350-003
